# The risk of believing that emotions are bad and uncontrollable: association with orthorexia nervosa

**DOI:** 10.1007/s40519-024-01710-3

**Published:** 2025-01-18

**Authors:** L. Vuillier, M. Greville-Harris, R. L. Moseley

**Affiliations:** Faculty of Science and Technology, Department of Psychology, Bournemouth University, Poole, UK

**Keywords:** Orthorexia nervosa, Eating disorder, Emotion regulation, Beliefs, Controllability

## Abstract

**Purpose:**

This study aimed to explore emotional functioning in individuals with varying levels of orthorexia nervosa (ON) symptoms. Given the established links between emotion dysregulation and other eating disorders (EDs), and the conceptualization of ON within the ED spectrum, this research sought to examine the relationships between ON symptomatology and emotion regulation strategies, alexithymia, and beliefs about emotions.

**Methods:**

A large sample (N = 562) completed self-report measures with high psychometric properties, assessing ON traits (E-DOS), emotion regulation strategies (DERS-SF and ERQ), alexithymia (TAS-20), and beliefs about emotions (ERQ). The study used well-validated measures to address limitations of previous research.

**Results:**

Individuals with high ON traits demonstrated difficulties in most aspects of emotional functioning compared to those with low ON traits. Suppression, but not reappraisal, partially mediated the relationship between beliefs about emotions and ON symptoms. Believing emotions are bad or useless, difficulty controlling impulses, and relying on suppression to regulate emotions were most strongly associated with ON symptoms.

**Conclusion:**

This study provides evidence that emotion dysregulation plays an important role in ON symptomatology. The findings suggest that when emotions feel unhelpful or uncontrollable, and maladaptive strategies like suppression are employed, individuals may seek perceived control through pathologically 'healthy' eating. There is currently no diagnosis criteria for ON, and consequently no clear treatment pathway. Our research suggests that specific aspects of emotional functioning such as beliefs about the usefulness of emotions or difficulties with feeling out of control when upset may be a useful treatment target to help individuals with ON develop healthier coping mechanisms and reduce reliance on rigid dietary rules as a means of emotional regulation.

Level of evidence.

Level III: Evidence obtained from well-designed cohort or case–control analytic studies.

**Supplementary Information:**

The online version contains supplementary material available at 10.1007/s40519-024-01710-3.

## Introduction

The pursuit of health and wellness has become a prevalent cultural norm, often characterized by an emphasis on healthy dietary practices aimed at achieving optimal physical well-being [1–3]. Within this backdrop, the concept of orthorexia nervosa (ON) has emerged as a subject of increasing concern. Defined as a pathological fixation on consuming "healthy" or "pure" foods, ON represents a complex interplay of dietary behaviours, psychological processes, and cultural factors [4–7]. Despite not yet being recognised in the Diagnostic and Statistical Manual of Mental Disorders, fifth edition (DSM-5) [8], or in the International Classification of Diseases, Eleventh Revision (ICD-11) [9], accumulating evidence suggests that ON resembles other eating disorders (ED) in its clinical presentation and psychological correlates, and should be conceptualised within that spectrum [10–12]. Proposed criteria for ON for example include a strong preoccupation with one's eating behaviour and with self-imposed rigid and inflexible rules, an obsession with “healthy” or “pure” eating, feelings of emotional distress if healthy food rules is violated, and with the adherence to these self-imposed dietary rules having an undue influence on their self-evaluation [13]. Prevalence rates vary greatly, partly due to the lack of clear diagnostic criteria and reliable measuring tools, but ON is estimated to affect about 6.9% of the general population [14]. Despite the growing interest in understanding the psychological underpinnings of ON, there remains a paucity of research examining its relationship with various facets of emotional functioning, including emotion regulation, beliefs about emotions, and alexithymia. This is however important because insights into emotional functioning could help better understand the mechanisms involved in the development and maintenance of ON, and potentially inform the development of new treatment strategies for ON.

Differences in emotional functioning are an integral part of ED. Emotion regulation difficulties have been identified as a transdiagnostic feature of eating psychopathology [15–18], as has alexithymia, a difficulty identifying and describing one’s emotions [19–21]; moreover, there is evidence that such difficulties precede and contribute to the development and maintenance of ED [22–24]. People with ED also report difficulties using adaptive emotion regulation strategies like cognitive reappraisal [15, 25, 26] and instead report more use of suppression as a way of regulating their emotions. Recent work has also highlighted the relevance of the beliefs people have about their emotions, such as the extent to which they are controllable, and whether they are useful—which also encompasses the extent to which they are helpful vs harmful [27–29]. Beliefs about the controllability of emotions are thought to influence and interact with the implementation of emotion regulation [28, 30]. For example, reduced belief in the controllability of emotions was found to predict less use of reappraisal and more use of suppression to regulate unpleasant emotions, and through this, higher levels of eating psychopathology [25]. Although belief about their *usefulness* per se remains unexplored in relation to eating disorders, beliefs about the unacceptable or threatening nature of emotions have been likewise linked to eating psychopathology [17, 31, 32], as well as with greater reactivity to stressors, lower well-being, and other aspects of psychopathology [33, 34]. In part, such beliefs may similarly increase the likelihood that individuals select less adaptive ER strategies they believe will help them *avoid* these emotions, such as emotional suppression.

While ON is conceptualised within the taxonomy of ED [10, 12], very little is presently known about the emotional functioning of individuals with ON, a paucity which may be all the more significant if emotional functioning plays as pivotal a role in its development and maintenance. Though no studies to date have examined the emotion beliefs of individuals with ON, Vuillier and colleagues [35] examined the relationship between orthorexic tendencies and difficulties with emotion identification and regulation. They found that individuals with higher levels of orthorexic tendencies reported greater difficulties in identifying and regulating their emotions, a finding that was supported by two other studies in Lebanese participants [36] and in adolescent athletes [37] The latter also found that their athlete sample reported less use of reappraisal and more use of suppression compared to those who did not participate in sport. Unfortunately, all three papers used versions of the ORTO-15 [38], a scale that has now been demonstrated to have poor psychometric properties such as low internal consistency, poor construct validity, and inconsistent factor structure across different populations [39, 40]. The ORTO-15 has also been accused of over estimating prevalence rates due to not measuring the marked distressed experienced by people with ON, leading to a high percentage of falsely positive results [41].The strength of these concerns in the scientific community is such that Barrada and Meule [40] repudiate the findings of any study using the scale. Consequently, very little is known about the emotional functioning of this population.

The current study aimed to explore patterns of emotional functioning associated with ON, using a well-validated measure of ON symptomatology. As ON is presently unrecognised in diagnostic criteria and so not consistently diagnosed by clinicians, the current study examined emotional functioning in relation to ON symptomatology in the general population (n = 562), a number of whom exhibited likely ON (n = 93). Participants completed self-report measures assessing their emotion regulatory strategies, levels of alexithymia and beliefs about emotions, as well as ON traits. We made the following hypotheses:

1. Based on previous research [35–37] and the prominence of emotion difficulties in ED [15–20, 25] we hypothesised that the people high in ON traits would show more difficulties regulating their emotions, higher alexithymia scores, less use of reappraisal and more use of suppression, as well as stronger beliefs about the uncontrollability and uselessness of emotions compared to people low in ON traits (H1).
2. As per relationships between beliefs in emotion controllability and ED psychopathology [25], we predicted a similar relationship to operate in ON. Specifically, we predicted that more use of suppression and less use of reappraisal would mediate the relationship between emotion controllability and ON symptomatology (H2a). Given the relevance of beliefs in the acceptability of emotions to psychopathology [17, 31], we likewise speculated a relationship between beliefs about the usefulness of emotions and levels of ON symptomatology (H2b).
3. Finally, in an exploratory analysis, we examined which aspects of emotional functioning (emotion regulation, levels of alexithymia and beliefs about emotions) were more closely related to ON symptomatology.

## Methods

### Participants

A G-power calculation showed that 369 participants were needed to have enough statistical power to observe a minimal 5% deviation of R^2^ from zero, a 5% risk of error, 80% power, and 13 factors to be entered in the multivariate analyses. We therefore recruited 583 participants to ensure a large enough sample after accounting for missing data. We excluded 21 participants from the data set if there were missing responses in one or more questionnaires or if the two attention check questions were not answered correctly.

The final sample included 562 participants (sex at birth: n = 412 females, n = 148 males, n = 2 other) with a mean age of 21.7 (SD = 7.3, age range = 57). The majority of participants were White British or White European (84.9%), the rest being mixed race ethnic groups (6.6%), Black (3.4%), Asian (2.8%), or other (2.3%). A total of 34 participants reported ever receiving a diagnosis of an eating disorder, with n = 21 reporting an active disorder at the time of the study, and n = 13 a history of an eating disorder. Most participants were students recruited from the lead author’s institution, or through word of mouth. Participants were told this was a study on emotions and mental health but the advert did not mention eating disorders or healthy eating specifically.

### The Dusseldorf orthorexia scale- english version (E-DOS)

The E-DOS [42] is a 10-item scale measuring orthorexic eating tendencies. Items are scored on a 4-point Likert scale from 1 (this does not apply to me) to 4 (this applies to me). Higher scores indicate greater ON symptomatology, with scores ≥ 25 indicating risk of ON (n = 93, or 16.5% of the sample) and ≥ 30 indicating likely presence of ON (n = 27, or 4.8% of the sample; similar to rates seen in other studies [14]. The E-DOS has good psychometric properties [42, 43] including excellent internal consistency which was confirmed in our sample (α = 0.87). We used the cut-off score of 25 to split our sample in a ‘at risk or likely presence of ON’ group and a ‘low ON’ group.

### Emotional regulation questionnaire (ERQ)

The ERQ [44] evaluates the frequency of use of reappraisal and suppression as strategies to regulate emotions. The scale consists of 10 questions (six for reappraisal and four for suppression) with responses scored on a 7-point scale from 1 (strongly disagree) to 7 (strongly agree). Scores for reappraisal range from 6 to 42, while those for suppression range from 4 to 28; in each, higher scores mean higher usage of that strategy. The ERQ has good psychometric properties [45] which was confirmed in our sample (reappraisal, α = 0.87; suppression, α = 0.76).

### The difficulty in emotion regulation scale, short form (DERS-SF)

The DERS-SF [46] is a 18-item self-report measure assessing clinical impairments in emotion regulation. It contains six subscales: lack of emotional clarity, lack of emotional awareness, difficulties engaging in goal-directed behaviour when upset, difficulties with impulse control when upset, non-acceptance of emotions, and limited access to emotion regulation strategies (henceforth ‘clarity’, ‘awareness’, ‘goals’, ‘impulse’, ‘non-acceptance’, ‘strategies’). Its items are scored on a 5-point scale from 1 (almost never) to 5 (almost always), with higher scores (ranging between 18 and 90) indicating more difficulties. This short-form has strong psychometric properties [47], which was confirmed in our sample (α = 0.91 for the total score; α = 0.82 for clarity; α = 0.76 for awareness; α = 0.89 for goals; α = 0.90 for impulse; α = 0.83 for non-acceptance; α = 0.82 for strategies).

### Emotion beliefs questionnaire (EBQ)

The EBQ [29] is a 16-item measure assessing beliefs about the controllability and goodness/usefulness of emotions in two subscales. Items are answered on a 7-point Likert scale from 1 (strongly disagree) to 7 (strongly agree). Scores ranging between 8 and 56, higher scores on the controllability subscale indicate that respondents believe that emotions are uncontrollable. Scores similarly ranging between 8 and 56, higher scores on the usefulness/goodness subscale indicates that respondents believe that emotions are bad and/or useless. It has good psychometrics properties [48] which was confirmed in our sample (α = 0.88 for the total score; α = 0.86 for controllability; α = 0.81 for usefulness).

### The toronto alexithymia scale (TAS-20)

The TAS-20 [49] has 20 statements which can be subdivided into three factors, reflecting difficulties identifying feelings (DIF), difficulties describing feelings (DDF), and an inclination towards externally-orientated thinking (EOT). Items are scored on a 5-point Likert scale from 1 (strongly disagree) to 5 (strongly agree). With scores ranging from 20 to 100, a total score of 61 or above indicates a clinically-substantive level of impairment (30.1% of the total sample; 43.0% of the ‘at risk of ON’ group; and 27.5% of the ‘low ON’ group). The TAS-20 has good psychometric properties including excellent internal consistency [50] which was confirmed in our sample (α = 0.84 for the total score; α = 0.86 for DIF; α = 0.79 for DDF; α = 0.54 for EOT—below the 0.70 threshold but not unusual for the EOT subscale [51].

### Procedure

The questionnaires were presented through the online platform Qualtrics (Qualtrics, Provo, UT) and began with demographic questions, followed by the DERS-SF, the TAS-20, the ERQ, the EBQ and the E-DOS. Participants also answered other questionnaires not reported in the current paper. This study received ethical approval from the Research Ethics Panel at the first author’s institution.

### Statistical analyses

To study the emotional functioning of people with ON symptoms (H1), we first performed a MANOVA on 13 dependent variables (DVs): the three alexithymia subscales (DIF, DDF, EOT), the six DERS subscales and total score (strategies, non-acceptance, impulse control, goals, awareness, clarity), and the two ERQ subscales (reappraisal and suppression), and the two EBQ subscales (controllability and usefulness). ON traits was our independent variable (IV) such that participants scoring below 25 were categorised in the low ON group (n = 469), and participants scoring 25 or above categorised in the high ON group (n = 93), as per the E-DOS. There were no gender (X^2^ (2, N = 562) = 2.46, p = 0.292), ethnicity (X^2^ (4, N = 562) = 2.12, p = 0.714), or age (M_high ON_ = 21.8 (7.2), M_low ON_ = 21.5 (7.5), t (522) = 0.30, p = 0.762) differences between the two groups. The alpha level was corrected to p = 0.004 to account for 13 comparisons (DVs). We also ran a supplementary analysis using a median split for a more even distribution of the sample, such that participants scoring 17 or below were categorised in the low ON group (n = 301, 53.3% of the sample), and participants scoring above 17 in the high ON group (n = 261, 46.2% of the sample).

To better understand the relationship between beliefs about emotions and ON symptoms, we ran two mediation analyses using the PROCESS [52] macro for SPSS (model 4), as it uses bootstrapping which resampled the data set 20,000 times. The first analysis (H2a) looked at beliefs about emotional controllability (Y) and the second analysis (H2b) looked at beliefs about the usefulness of emotions (Y), on symptoms of orthorexia (Y) via reappraisal (M1) and suppression (M2).

To determine which aspect of emotional functioning were most closely related to ON symptomatology (H3), we ran a linear regression with all 13 variables of emotional functioning as predictors and ON symptoms as outcome. We used the backward method to remove all non-significant predictors. All VIF scores were well below 5 (maximum VIF was 2.8) and the tolerance scores above 0.2 (minimum tolerance was 0.4), showing no multicollinearity between the variables.

## Results

### H1: Comparing participants at risk of ON vs low in ON traits on a range of measures of emotional functioning

As shown in Table 1, significance tests for our dependent variables showed that our participants at risk of or with likely ON reported more difficulties with their emotions compared to our participants low in ON traits (F (13, 547) = 3.29, p < 0.001, η^2^ = 0.07). Specifically, they showed more difficulties identifying and describing their emotions, less emotional clarity and acceptance, and more difficulties controlling impulses and accessing strategies when upset. They also showed more use of suppression and more beliefs that emotions are uncontrollable and are useless, compared to participants low in ON traits. These results were replicated using the median split (see supplementary material).

**Table 1:** Comparing low vs high ON in emotional functioning

	Mean (SD)		High vs low ON symptoms		
	*Low ON traits*	*At risk or likely ON*	*F*	*p*	η^2^
TAS					
DIF	18.5 (6.5)	20.9 (5.7)	**10.6**	**0.001**	**0.02**
DDF	15.2 (4.7)	16.4 (4.0)	5.4	0.021	0.01
EOT	19.4 (4.3)	20.5 (4.4)	5.1	0.024	0.01
DERS					
Strategies	8.0 (3.1)	9.2 (3.4)	**10.8**	**0.001**	**0.02**
Non-acceptance	8.5 (3.3)	9.5 (3.3)	***7.5***	***0.006***	**0.01**
Impulse	6.8 (3.3)	8.5 (4.0)	***19.7***	** < *.001***	***0.03***
Goals	10.7 (3.2)	11.4 (3.1)	*3.9*	*0.049*	*0.01*
Awareness	7.2 (2.6)	7.8 (2.8)	*4.2*	*0.042*	*0.01*
Clarity	7.5 (2.7)	8.6 (2.7)	***12.0***	** < *.001***	***0.02***
ERQ					
Reappraisal	26.1 (7.0)	25.4 (7.6)	*0.6*	*0.448*	*0.00*
Suppression	15.0 (5.1)	16.8 (4.9)	***10.2***	***0.001***	***0.02***
EBQ					
Controllability	21.5 (8.3)	25.5 (9.2)	***17.4***	*** < 0.001***	***0.03***
Usefulness	18.2 (6.9)	21.8 (8.2)	***19.5***	*** < 0.001***	***0.03***

Bold font shows significant differences after Bonferroni correction adjusting for 13 comparisons (p = .004)

### H2: The mediating role of suppression and reappraisal on the relationship between beliefs about emotions and ON traits

**Fig. 1:**
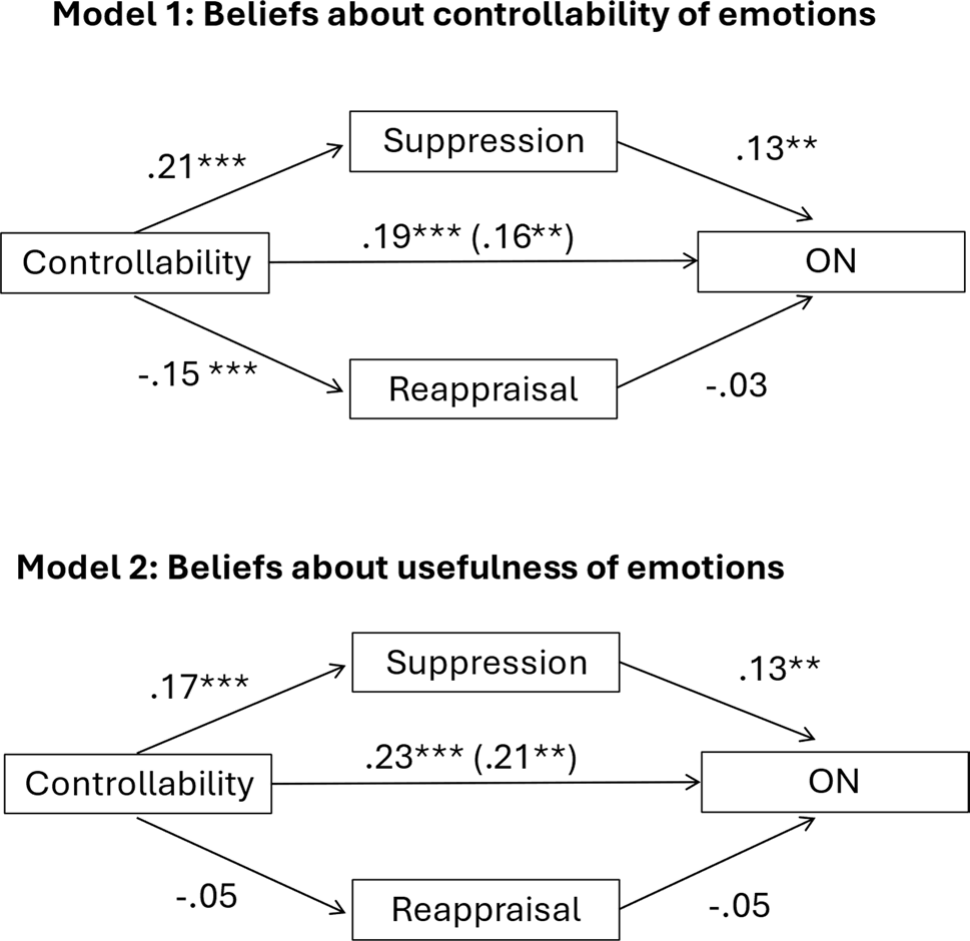
Mediation models representing the relationship between beliefs about emotional controllability (model 1) or beliefs about the usefulness of emotions (model 2) and ON symptoms, mediated via suppression but not reappraisal. *** denotes < 0.001 significance, ** denote < 0.01 significance

The results for the mediation analysis on emotional *controllability* (H2a) and Model 2 on beliefs about the *usefulness* of emotions (H2b) are displayed in Fig. 1.

As predicted (H2a), there was a significant negative relationship between beliefs about emotional controllability and ON psychopathology (path c: *β* = 0.19, *p* < 0.001). Beliefs about emotional uncontrollability were associated with more use of suppression (path a1:* β* = 0.21, *p* < 0.001) as well as less use of reappraisal (path a1:* β* = − 0.15, *p* < 0.001). ON psychopathology was associated with more use of suppression (path b1:* β* = 0.13, *p* = 0.002), but not less use of reappraisal (path b2:* β* = − 0.03, *p* = 0.443). When controlling for the mediating variable of suppression and reappraisal, the direct effect of beliefs about emotional controllability on ON symptoms remained significant but was reduced suggesting partial mediation (path c’:* β* = 0.16, *p* = 0.003). The indirect effect of beliefs about emotional controllability on ON symptoms via greater use of suppression was significant (*ab* = 0.02, CI 0.001, 0.006), but the indirect effect via reappraisal was not (*ab* = 0.004, CI − 0.006, 0.014).

As predicted (H2b), there was also a significant negative relationship between beliefs about the usefulness of emotions and ON psychopathology (path c: *β* = 0.23, *p* < 0.001). Beliefs about the uselessness of emotions were associated with more use of suppression (path a1:* β* = 0.17, *p* < 0.001) but not less use of reappraisal (path a1:* β* = − 0.05, *p* = 0.291). ON psychopathology was associated with more use of suppression (path b1:* β* = 0.13, *p* = 0.002), but not less use of reappraisal (path b2:* β* = − 0.05, *p* = 0.262). When controlling for the mediating, the direct effect of beliefs about the usefulness of emotions on ON symptoms remained significant but was reduced suggesting partial mediation (path c’:* β* = 0.21, *p* < 0.001). The indirect effect of beliefs about emotional usefulness on ON symptoms via greater use of suppression was significant (*ab* = 0.02, CI 0.007, 0.035), but the indirect effect via reappraisal was not (*ab* = 0.002, CI − 0.002, 0.009).

### H3: Which aspects of emotional functioning contribute most to ON symptomatology?

We found that beliefs about usefulness of emotions (β = 0.19, p < 0.001), difficulties controlling impulse (β = 0.18, p < 0.001) and relying on suppression to regulate emotions (β = 0.11, p = 0.007) were the only statistically significant factors associated with ON symptoms and explained 10% of the variance of symptoms of orthorexia nervosa in our sample (R^2^ = 0.1, F (3,557) = 20.4, p < 0.001).

## Discussion

This study aimed to assess the emotional functioning of people with various levels of ON. Whilst previous research was suggestive that people high in ON symptoms might have difficulties identifying and regulating their emotions [35–37], these findings were cast into doubt by the poor psychometric properties of the ORTO questionnaire used [39, 40]. Moreover, no research has looked at the effect of beliefs about the usefulness and controllability of emotions in ON. We used a large sample (N = 562) and questionnaires with strong psychometric properties to first confirm difficulties with emotions in people high vs low in ON traits (H1); second, to understand the mechanisms underlying the relationship between beliefs about emotions and ON (H2); and third, to understand which aspect of emotional functioning is most associated with ON (H3). We confirmed difficulties on most variables of emotional functioning in people with high ON traits compared to participants low in these traits. We also found that suppression, but not reappraisal, partially mediated the relationship between beliefs about emotions and ON symptoms. Finally, we found that believing that emotions are bad or useless, as well as difficulties controlling impulse, and relying on suppression to regulate emotions, were most strongly associated with ON symptoms. We discuss our results in turn.

Our study, which used questionnaires with strong psychometric properties, was supportive of the previous findings suggesting that people with high ON symptoms have difficulties identifying and regulating their emotions [35–37]. Whilst our design does not allow us to identify whether such difficulties are a cause or a consequence of ON, it is interesting to note similarities with other EDs such as AN and BN, for which emotional difficulties play a role in both their maintenance and development [15, 16, 19, 22–24]. Interestingly however, the use of reappraisal was not linked to ON symptomatology. Research has shown that people with EDs such as AN, BN and BED do not tend to use cognitive reappraisal to regulate emotions [15, 25, 26]. D’Urso and colleagues [37] also showed that their adolescent athletes used less reappraisal than their non athlete counterparts. However, we could not replicate these findings in our sample. While reappraisal is generally considered an adaptive strategy [44], its effectiveness is context-dependent. For instance, in problem gambling, high reappraisal has been associated with increased harm, possibly due to the reframing of risky behaviors as less dangerous [53–55]. In the context of ON, it is possible that individuals might use reappraisal to justify their focus on "healthy" eating as a means of managing distress, rather than addressing underlying emotional issues. Some of the ERQ items for reappraisal focus on changing thoughts to reduce negative emotions. It is possible that individuals with ON symptoms may indeed engage in this process, but by redirecting their thoughts towards their dietary practices rather than directly addressing emotional states. This could explain why we found no clear positive or negative relationship between reappraisal and ON symptoms. We recommend further research to elucidate whether reappraisal serves as a helpful coping mechanism or potentially reinforces maladaptive behaviors in this population.

This paper is the first to explore the role of beliefs about emotions in ON. We found that people high in ON traits were more likely to think their emotions uncontrollable and useless, and that the relationships between these beliefs and ON symptoms were mediated by the use of suppression; the more people held these beliefs, the more they used suppression, and the higher their orthorexic tendencies. Suppression does not actually change the emotional experience of a person, rather it changes the emotional expression (i.e. the person will still feel sad/anxious/upset) [44]. Therefore, it is possible that people with an interest in healthy eating who believe emotions to be useless and uncontrollable, may develop an unhealthy obsession with healthy food *as a way of* coping with unpleasant or difficult feelings. Interestingly, Gerges and colleagues [56] found that positive beliefs about worry, for example that worrying (i.e. the thought rather than the emotion) is good for you, were associated with high ON. We instead found that the belief that emotions are bad or useless was associated with high ON. It is important to note that their paper looked at beliefs about *thoughts* rather than *emotions*. So it is possible that people with high ON symptoms do believe that emotions are bad or useless, but that *thinking* about unpleasant emotions may be a way of managing them when they have no access to other strategies. The process of thinking about worry closely resembles rumination [57]. Although we did not measure rumination in our study, numerous studies have shown that individuals with eating disorders frequently employ this strategy [58, 59]. Future research should investigate the roles of beliefs about both emotions and thoughts, as well as their connections to emotion dysregulation in ON.

Our findings revealed that individuals with high ON symptoms tend to feel out of control when upset, possibly due to a perceived inability to manage their emotions. This loss of control may drive them to seek control through food intake regulation. Qualitative research supports this hypothesis, indicating that individuals with ON symptoms often use dietary rules as a coping mechanism to feel safe and in control [60, 61], and to manage health anxiety [7]. Recent studies have consistently shown a strong association between high ON symptomatology and elevated health anxiety. These individuals often believe they can control their health by regulating their food intake, especially when other life aspects feel unmanageable [7, 60–63]. This suggests that stringent dietary control may be implemented as a strategy to cope with health anxiety, particularly when individuals perceive low emotional controllability and struggle with emotion regulation. Paradoxically, this rigid dietary control often leads to increased emotional distress and feelings of loss of control, especially during dietary lapses [7, 61]. This cyclical pattern may perpetuate and exacerbate ON symptoms. Future research should explore the association between health anxiety, beliefs about emotional controllability and dietary control in ON.

This study has implications in terms of understanding the aetiology and potential treatment avenues for ON. Firstly, this work suggests that emotion dysregulation plays a role in ON. While it is unclear whether emotion dysregulation serves as a causal and/or maintaining factors in ON, it appears that individuals with ON may use their strict dietary rules as a means of coping with negative emotional experiences. This is similar to other EDs [15–20, 25] and suggests the importance of further investigation of the role of emotions when considering models and risk factors for ON symptomatology. Second, this study suggests useful avenues for treatment; specific aspects of emotion regulation such as beliefs about the usefulness of emotions, difficulties with feeling out of control when upset, and reliance on unhelpful strategies such as suppression, were predictors of ON symptomatology. In targeting these aspects, clinicians may help individuals with ON develop healthier coping mechanisms and reduce reliance on rigid dietary rules as a means of emotional regulation. In line with other ED treatment models [64, 65], integrating emotion-focused interventions when developing treatment approaches for ON may enhance treatment outcomes and reduce the risk of relapse.

## Strengths and limitations

While the present study demonstrates relationships between emotion difficulties and ON psychopathology akin to those observed more broadly in ED [15–20, 25], directional designs are necessary to confirm whether differences such as the ones we observed are causal factors in the development and maintenance of ON. Presently, a strong conceptual understanding of ON is hampered by lack of clinical and scientific consensus around its definition [6, 13], and its subsequent exclusion from diagnostic manuals. Clinicians cannot formally give diagnoses of ON, but also differ to the extent of their willingness to informally identify individuals as suffering from ON [66–68]. As such, recruiting participants on the basis of an informal diagnosis entails inherent unreliability. We approached this problem by examining ON symptomatology as distributed in the general population, circumventing the need for clinical diagnoses, but this meant that we could not validate the clinical status of those who did fall above the likely ON threshold on the E-DOS.

Our sample was limited in way of ethnic, sex and gender diversity, and findings may not be generalised to manifestations of ON in other cultures [69]. While ethnicity influences the symptom presentation and treatment needs of individuals with ED [70], as well as emotional function [71], it is rarely collected in ON research to date [12]. While we collected this information about participants, under-representation of ethnic minorities precluded any conclusions being drawn on this factor. Similarly, while sex and gender influence presentation in people with ED [72, 73] and those with ON [74], our predominantly cisgender female sample meant that we were unable to examine potential sex or gender differences in emotional functioning. This is a relevant line of enquiry given that emotion differences may be differentially related to ED symptomatology in men and women [26].

Importantly, whilst this study suggests that the role of controlling outwards signs of emotions and difficulties controlling impulse may be important in ON, the use of self-report measures means that it is difficult to discern whether this is subjective or objective. Indeed, other pathological eating behaviours, particularly in AN, are linked to maladaptive overcontrol [75], and it is possible that such rigid and perfectionistic beliefs about control are present in ON. If so, this could lead to over-inflation of *perceived* lack of control, rather than an observable lack of impulse control per se. Future research should use more objective measure to better understand whether people with ON do act impulsively when upset, and suppress outwards sign of emotions.

## What is already known on this subject?

Plenty of research highlights the role of different facets of emotional functioning in ED pathology [15–20, 25]. However, while there is growing research into the psychological underpinnings of ON [4–7], there is a paucity of research examining the role of facets of emotions in ON-related eating pathology. This is important because understanding the role of emotions has important implications for understanding the aetiology and treatment of ON.

## What this study adds?

This study indicates that emotion dysregulation plays an important role in ON symptomatology. Beliefs that emotions are not useful, difficulty controlling impulses and reliance on suppression to cope with emotions were the strongest predictors of ON. In particular, we postulate that when emotions feel unhelpful or ‘uncontrollable’, and when maladaptive strategies such as suppression are employed, perceived control may be sought through the pursuit of pathologically ‘healthy’ eating. This adds to our current understanding of ON and highlights the potential for drawing upon ED emotion-focussed models when developing treatment interventions for ON symptomatology.

## Supplementary Information

Below is the link to the electronic supplementary material.

Supplementary file 1.

## Data Availability

The datasets generated during and/or analysed during the current study are available on BORDaR (https://doi.org/10.18746/bmth.data.00000389) or from the corresponding author.
