# Correction: The risk of believing that emotions are bad and uncontrollable: association with orthorexia nervosa

**DOI:** 10.1007/s40519-025-01750-3

**Published:** 2025-05-28

**Authors:** L. Vuillier, M. Greville-Harris, R. L. Moseley

**Affiliations:** Faculty of Science and Technology, Department of Psychology, Bournemouth University, Poole, UK

**Correction: Eating and Weight Disorders – Studies on Anorexia, Bulimia and Obesity (2025) 30:8** 10.1007/s40519-024-01710-3

In this article [1], the wrong figure appeared as Fig. 1; the second model on that Figure should say usefulness rather than controllability.

For completeness and transparency, the old incorrect and correct versions are displayed below.

Incorrect Fig. 1

**Fig. 1:**
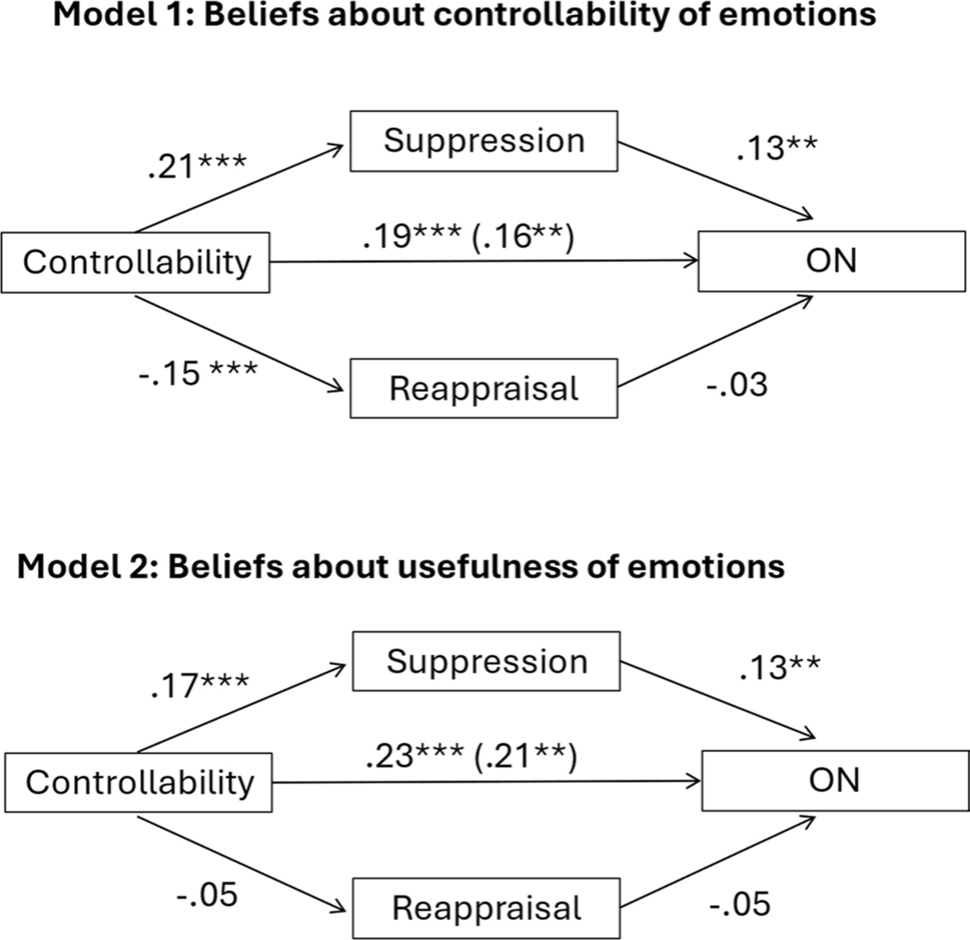
Mediation models representing the relationship between beliefs about emotional controllability (model 1) or beliefs about the usefulness of emotions (model 2) and ON symptoms, mediated via suppression but not reappraisal. *** denotes < 0.001 significance, ** denote < 0.01 significance

Correct Fig. 1

**Fig. 1:**
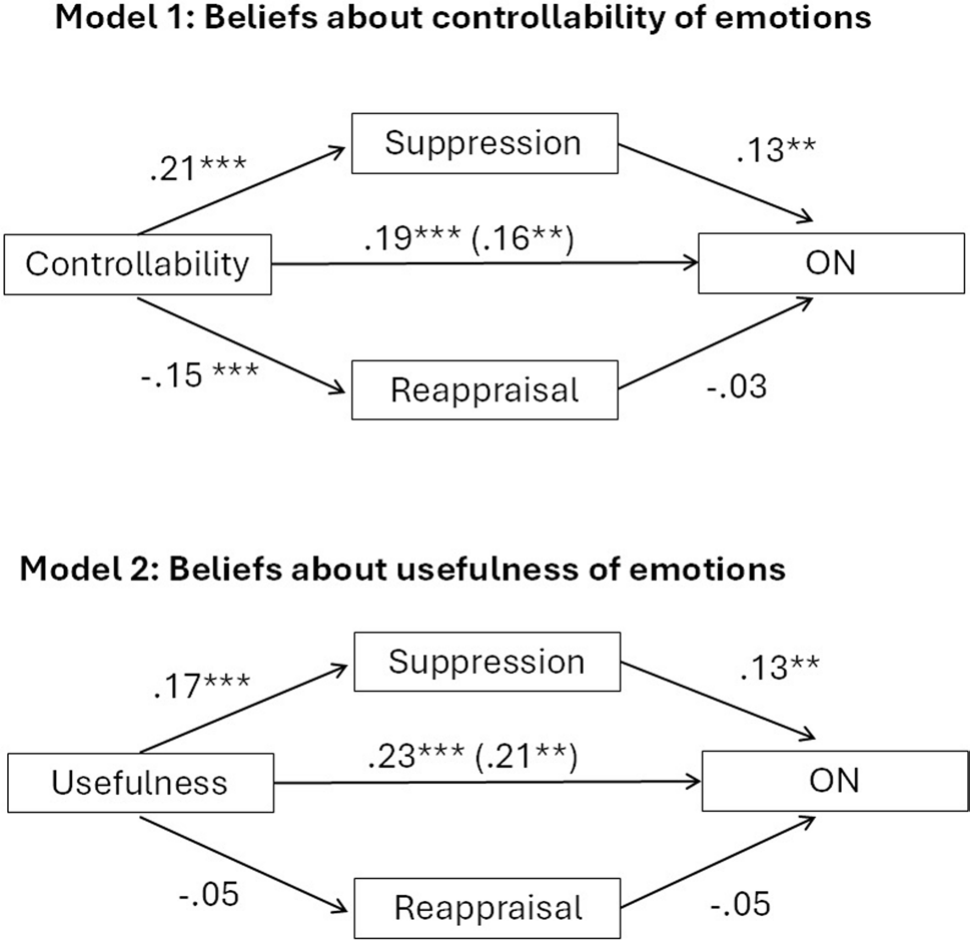
Mediation models representing the relationship between beliefs about emotional controllability (model 1) or beliefs about the usefulness of emotions (model 2) and ON symptoms, mediated via suppression but not reappraisal. *** denotes < 0.001 significance, ** denote < 0.01 significance
