# Supplementary material for: The risk of believing that emotions are bad and uncontrollable: association with orthorexia nervosa
Source: Eat Weight Disord. 2025 Jan 18;30(1):8. doi: 10.1007/s40519-024-01710-3 (PMC11742882; doi:10.1007/s40519-024-01710-3)
Supplement: Supplementary file 1 [file 40519_2024_1710_MOESM1_ESM.docx]

**Table S1: Comparing high ON vs low ON symptoms in emotional functioning, based on a median split on the E-DOS (low ON for a score of 17 or below; high ON for a score above 17)**

|  | | Mean (SD) | | High vs low ON traits | | |
| --- | --- | --- | --- | --- | --- | --- |
|  | *Low ON traits* | | *High ON traits* | *F* | *p* | η^2^ |
| **TAS** |  | |  |  |  |  |
| DIF | 17.8 (6.5) | | 20.1 (6.2) | **17.8** | **<.001** | **.03** |
| DDF | 14.9 (4.7) | | 16.0 (4.5) | 8.0 | **.005** | .01 |
| EOT | 19.3 (4.3) | | 20.0 (4.2) | 3.1 | .077 | .01 |
|  |  | |  |  |  |  |
| **DERS** |  | |  |  |  |  |
| Strategies | 7.8 (3.1) | | 8.7 (3.2) | **13.0** | **<.001** | **.02** |
| Non-acceptance | 8.1 (3.3) | | 9.2 (3.2) | ***13.6*** | ***<.001*** | **.02** |
| Impulse | 6.5 (3.2) | | 7.7 (3.7) | ***18.9*** | ***<.001*** | ***.03*** |
| Goals | 10.6 (3.3) | | 11.1 (3.2) | *4.0* | *.047* | *.01* |
| Awareness | 7.1 (2.6) | | 7.3 (2.7) | *2.5* | *.113* | *.00* |
| Clarity | 7.3 (2.7) | | 8.1 (2.8) | ***11.2*** | ***<.001*** | ***.02*** |
|  |  | |  |  |  |  |
| **ERQ** |  | |  |  |  |  |
| Reappraisal | 26.1 (7.2) | | 25.8 (7.0) | *0.3* | *.586* | *.00* |
| Suppression | 14.5 (5.1) | | 16.1 (5.0) | ***14.7*** | ***<.001*** | ***.03*** |
|  |  | |  |  |  |  |
| **EBQ** |  | |  |  |  |  |
| Controllability | 20.7 (8.4) | | 23.9 (8.5) | ***20.5*** | ***<.001*** | ***.04*** |
| Usefulness | 17.3 (6.3) | | 20.5 (7.8) | ***29.0*** | ***<.001*** | ***.05*** |
